# Blood Plasma of Patients with Parkinson's Disease Increases Alpha-Synuclein Aggregation and Neurotoxicity

**DOI:** 10.1155/2016/7596482

**Published:** 2016-11-14

**Authors:** Peng Wang, Xin Li, Xuran Li, Weiwei Yang, Shun Yu

**Affiliations:** ^1^Department of Neurobiology, Xuanwu Hospital of Capital Medical University, Beijing, China; ^2^Department of Human Anatomy, School of Basic Medical Sciences, Beihua University, Jilin, China; ^3^Center of Parkinson's Disease, Beijing Institute for Brain Disorders, Beijing, China; ^4^Beijing Key Laboratory for Parkinson's Disease, Beijing, China

## Abstract

A pathological hallmark of Parkinson's disease (PD) is formation of Lewy bodies in neurons of the brain. This has been attributed to the spread of *α*-synuclein (*α*-syn) aggregates, which involves release of *α*-syn from a neuron and its reuptake by a neighboring neuron. We found that treatment with plasma from PD patients induced more *α*-syn phosphorylation and oligomerization than plasma from normal subjects (NS). Compared with NS plasma, PD plasma added to primary neuron cultures caused more cell death in the presence of extracellular *α*-syn. This was supported by the observations that phosphorylated *α*-syn oligomers entered neurons, rapidly increased accumulated thioflavin S-positive inclusions, and induced a series of metabolic changes that included activation of polo-like kinase 2, inhibition of glucocerebrosidase and protein phosphatase 2A, and reduction of ceramide levels, all of which have been shown to promote *α*-syn phosphorylation and aggregation. We also analyzed neurotoxicity of *α*-syn oligomers relative to plasma from different patients. Neurotoxicity was not related to age or gender of the patients. However, neurotoxicity was positively correlated with H&Y staging score. The modification in the plasma may promote spreading of *α*-syn aggregates via an alternative pathway and accelerate progression of PD.

## 1. Introduction

Parkinson's disease (PD) is a progressive neurodegenerative disorder characterized pathologically by intracellular protein inclusions in specific neurons of the brain. These inclusions, which are known as Lewy bodies (LBs) and Lewy neurites [1], are collectively referred to as Lewy pathology. The major component of LBs is fibrillary alpha-synuclein (*α*-syn), a 140-amino-acid protein normally present in neurons in a monomeric form [2]. Before forming fibrils, *α*-syn aggregates into oligomers and protofibrils, which are the toxic species that cause neurodegeneration [3–5]. Although many factors promote *α*-syn aggregation in experimental studies [6–8], phosphorylation at Ser 129 may be the major factor that induces *α*-syn aggregation under pathological conditions; 90% of *α*-syn in LBs is phosphorylated at Ser129 compared with ≤4% in normal brains [9]. The role of phosphorylation in *α*-syn aggregation was confirmed by* in vitro* studies demonstrating that phosphorylated *α*-syn showed increased formation of soluble oligomers [6, 8, 10]. In addition, enzymes regulating *α*-syn phosphorylation undergo changes in expression and activity in brains with LB pathology. For example, polo-like kinase 2 (PLK2), which promotes *α*-syn phosphorylation, is upregulated in the brains of patients with Alzheimer's and LB disease [11, 12], while activity of protein phosphatase 2A (PP2A), which is responsible for *α*-syn dephosphorylation, is decreased in cases of dementia with LB and *α*-syn triplication [13, 14]. *β*-Glucocerebrosidase (GCase) is a lysosomal hydrolase that breaks down glucosylceramide (GlcCer) into glucose and ceramide [15]. GCase activity and ceramide levels are reduced in PD patient brains [16]. Since ceramide is a natural activator of PP2A [17], its reduction reduces PP2A activity, which indirectly promotes *α*-syn phosphorylation.

PD is characterized clinically by motor impairment that results from loss of dopaminergic neurons in the substantia nigra pars compacta [18]. It also manifests as various nonmotor symptoms, such as constipation, bradycardia, olfactory deficits, depression, and cognitive impairment [19, 20], which are associated with *α*-syn aggregation in extranigrostriatal regions of the central and peripheral nervous systems [21, 22] and are thought to result from prion-like spreading of *α*-syn aggregates [23, 24]. According to the Braak staging system, *α*-syn aggregation likely occurs in the olfactory bulb and/or the enteric nervous system and propagates between nerve cells [22]. In addition to possible transsynaptic transmission [25], an extracellular mechanism may promote the spread of *α*-syn pathology [26], which might involve release of *α*-syn from a cell, its uptake by a neighboring cell, and a pathological state induced in the recipient cell via seeding of *α*-syn monomers [27]. Indeed, both *α*-syn monomers and oligomers are released from neurons [28] and are present in extracellular fluids, including cerebrospinal fluid and blood plasma [29].* In vitro* and* in vivo* experiments have shown that these molecules are internalized by neurons via endocytosis or translocation across the plasma membrane [30]. Some types of *α*-syn oligomers can be induced to form aggregates [31], suggesting that these species are responsible for the spread of *α*-syn pathology. However, it is unknown whether extracellular *α*-syn monomers also play a role in this process.

We hypothesized that extensive *α*-syn pathology in PD patients may be influenced by changes in the internal metabolic environment that favor modification and aggregation of extracellular *α*-syn. To test this hypothesis, we cultured primary rat brain neurons in medium containing plasma from normal subjects (NS) or PD patients and evaluated phosphorylation and aggregation of *α*-syn and their relationships to cell viability.

## 2. Materials and Methods

### 2.1. Patients and Plasma Samples

PD patients and NS, which were matched in gender and age, were recruited for the study (Table 1). Inclusion criteria were idiopathic PD cases receiving l-dopa and/or dopamine treatment. Exclusion criteria were parkinsonism other than idiopathic PD, history of brain surgery/deep brain stimulation, or dementia as defined by the Diagnostic and Statistical Manual of Mental Disorders, 4th edition criteria. Mild cognitive impairment was not a criterion for exclusion.

Consecutive patients who met the UK Brain Bank Criteria for idiopathic PD were recruited through the Outpatient Departments at Xuanwu and Tiantan Hospitals, Capital Medical University, Beijing, China. Patients were tested during their “on” periods while on their standard drug regimen. All participants provided written informed consent for participation in the study, and the protocol was approved by the Ethics Committees of Xuanwu and Tiantan Hospitals.

Patients were examined by neurologists specializing in movement disorders using the motor subsection of the United Parkinson's Disease Rating Scale (UPDRS) [32]. None of the participants met the criteria for depression (assessed by the Beck Depression Inventory) or dementia (according to the Montreal Cognitive Assessment). The l-dopa equivalent daily dose was calculated for each patient as previously described. All tests were performed in Chinese.

Blood was collected in EDTA-coated vacuum tubes, and the plasma was separated by density gradient centrifugation at 400 ×g for 20 min. Plasma sample endogenous *α*-syn was removed by affinity purification using an anti-*α*-syn antibody. For each 10 mL plasma sample, 100 *μ*L Protein G with 50 *μ*g anti-*α*-syn antibody 3D5 (AB_2315782) [33] was added. After incubating 16 h at 4°C, each sample was centrifuged at 12000 ×g for 5 min. The plasma samples were then checked by enzyme-linked immunosorbent assay (ELISA), aliquoted, and stored at −80°C.

### 2.2. Primary Rat Cortical Neuron Cultures

Newborn Wistar rats were purchased from Beijing Vital River Laboratory Animal Co. (Beijing, China) and used within 24 h after birth. All animal experiments were conducted in accordance with the guidelines of the National Institutes of Health (Bethesda, MD, USA), and the study protocol was approved by the local Animal Care and Use Committee. Animals were killed by decapitation. Their brains were removed, and the bilateral cortices were dissected. Primary rat cortical neurons were dissociated according to a previously described method [34] and seeded in 96-well plates (Corning, Corning, NY, USA) at a density of 1 × 10^4^ cells/well or in 35-mm dishes (Corning) at a density of 1 × 10^5^ cells/cm^2^. The plates and dishes were coated with poly-d-lysine (Carolina Biological Supply, Burlington, NC, USA). Cells were cultured in Dulbecco's modified Eagle's medium (DMEM; Sigma-Aldrich, St. Louis, MO, USA) containing 10% horse serum (HyClone, Logan, UT, USA), 10% fetal calf serum (HyClone), 100 U/mL penicillin, and 100 *μ*g/mL streptomycin. On day 3, cultures were treated with 3 *μ*g/mL cytosine arabinoside (Sigma-Aldrich) to suppress growth of nonneuronal cells. Cells were allowed to grow for 14 d with regular medium changes.

### 2.3. Lentiviral (LV) Transfer Vector Construction and Transduction

LV vectors encoding green fluorescent protein- (GFP-) recombinant human *α*-syn fusion protein (LV-GFP-*α*-syn) (1 × 10^8^ transducing units) were constructed by Genechem (Shanghai, China). Primary neurons treated with cytosine arabinoside were infected with 2 *µ*L LV-GFP-*α*-syn or LV-GFP for 3 d. Recombinant human *α*-syn overexpression in infected neurons was evaluated by immunocytochemistry and western blotting.

### 2.4. Determination of Cell Viability and Apoptotic Cell Death

Cell viability was assessed with the Cell Titer-Blue Cell Viability Assay kit (Promega, Madison, WI, USA) according to the manufacturer's instructions. Apoptotic cell death was determined by flow cytometry using the annexin V-fluorescein isothiocyanate (FITC)/propidium iodide (PI) apoptosis detection kit (Yeasen, Shanghai, China). Primary neurons were washed with 0.01 M phosphate-buffered saline (PBS, pH 7.4), digested with 0.1% trypsin, and triturated with a Pasteur pipette. Cells were resuspended in 400 *µ*L binding buffer (1 × 10^5^ cells/mL). The cell suspension was incubated with 5 *µ*L annexin V FITC in the dark at 4°C for 15 min and with 10 *µ*L PI for 5 min. The rate of apoptosis was measured by flow cytometry (S3 Cell Sorter, Bio-Rad, Hercules, CA, USA).

### 2.5. Immunocytochemistry

Primary neurons were fixed with 4% paraformaldehyde and permeabilized with 0.1% Triton X-100 in PBS. Neurons were incubated in primary antibodies, monoclonal mouse anti-human *α*-syn antibody 3D5 (1 : 1000), polyclonal rabbit anti-human Ser129 *α*-syn antibody (1 : 500, Santa Cruz, CA, USA), or polyclonal mouse anti-ubiquitin (1 : 200; Abcam), followed by Alexa Fluor-conjugated secondary antibodies (Invitrogen, Carlsbad, CA, USA). For thioflavin S staining, cells were incubated with 0.05% thioflavin S (Sigma-Aldrich, St. Louis, MO, USA) in 0.01 M PBS for 8 min, washed for each 5 min 3 times with 80% EtOH, and then blocked for subsequent immunostaining. Different target proteins were distinguished by immunolabeling. Immunofluorescence was visualized by confocal microscopy (TCS-SP2; Leica, Heidelberg, Germany), and signal intensity was measured at the same background level and brightness/contrast settings. Image J software (http://rsbweb.nih.gov/ij/) was used for data analysis. All experiments were performed a minimum of 3 times.

### 2.6. Immunoprecipitation and Western Blot Analysis

Neurons were washed twice with Versene and 0.5% trypsin/EDTA to remove extracellular *α*-syn and then lysed in ice-cold lysis buffer composed of 50 mM Tris at pH 7.4, 150 mM NaCl, 0.1% Nonidet (N) P-40, and a protease inhibitor cocktail. For the insoluble inclusion assay, neurons were lysed in radioimmunoprecipitation buffer containing 1% Triton X-100 or 2% sodium dodecyl sulfate (SDS) and then boiled. After clearance by centrifugation at 10000 ×g for 15 min, 1 mg whole-cell lysate in lysis buffer was incubated with primary antibody overnight at 4°C. The beads were washed twice with buffer containing 500 mM NaCl and 0.5% NP-40 and then 3 times with lysis buffer. Immunoprecipitated proteins were resolved by 12.5% SDS polyacrylamide gel electrophoresis and blotted onto a polyvinylidene difluoride membrane (Millipore, Billerica, MA, USA). The membrane was then blocked in 5% bovine serum albumin in PBS and probed with primary antibodies followed by horseradish peroxidase-conjugated secondary antibody. Protein bands were visualized with the SuperSignal West chemiluminescence kit (Pierce, Rockford, IL, USA) according to the manufacturer's protocol.

### 2.7. Detection of Oligomeric and Phosphorylated *α*-syn by ELISA

Levels of *α*-syn oligomers or *α*-syn phosphorylated at Ser129 (pS-*α*-syn) in cultured neuron and brain tissue lysates were measured by ELISA as previously described [29, 35] but with nonbiotinylated and biotinylated 3D5 mouse monoclonal anti-*α*-syn antibodies (AB_2315782) or anti-Ser129 *α*-syn antibody (Santa Cruz, Dallas, TX, USA) for capture and detection, respectively. Serial dilutions of *α*-syn oligomer or pS-*α*-syn mixtures were used as standards. After completion of the immunoreaction, each well of the ELISA plate was incubated with 100 *µ*L ExtrAvidin alkaline phosphatase (Sigma-Aldrich, St. Louis, MO, USA) diluted 1 : 20000 in blocking buffer and reacted with p-nitrophenyl phosphate (Sigma-Aldrich, St. Louis, MO, USA). The reaction was incubated for 30 min at room temperature, and absorbance was read at 405 nm using a microplate reader (Multiskan MK3; Thermo Scientific, Waltham, MA, USA).

### 2.8. Phosphorylation Analysis by Sequential Window Acquisition of All Theoretical Mass Spectra (SWATH)

The *α*-syn incubated in culture medium containing NS or PD plasma was purified by immunoprecipitation. Ten *µ*g of 3D5 anti-*α*-syn antibody and 50 *µ*L of Protein G Sepharose Fast Flow (GE, San Diego, CA, USA) were incubated with 500 *µ*L of *α*-syn-containing medium at 4°C for 24 h. Next, the mixture was centrifuged at 1500 ×g for 5 min at 4°C. The pellet was washed several times with IP buffer (10 mM Tris-Cl at pH 7.5, 150 mM NaCl, 2 mM EDTA, and 0.5% Triton X-100), and purity was assessed by western blotting.

The immunoprecipitated *α*-syn samples were analyzed by liquid chromatography-tandem mass spectrometry (MS/MS) on a Triple TOF 5600 Plus system (Ab Sciex, Framingham, MA, USA) in 2 phases, data-dependent acquisition (DDA) followed by SWATH acquisition of the same sample, with the same gradient conditions and sample amounts. The original MS/MS data generated by DDA were analyzed with Protein Pilot v.4.5 software against the Cricetidae component of the Uniprot database. Spectral library generation and SWATH data processing were performed using Skyline v.2.5 software. A spectral library document was automatically generated prior to target data extraction.

### 2.9. GCase Activity Assay

GCase activity in the sample was determined using the QuantiChrom *β*-Glucosidase Assay kit (BioAssay Systems, Hayward, CA, USA). Distilled water (20 *μ*L) was added to 2 wells of a clear bottom 96-well plate. Next, 200 *μ*L of distilled water or calibrator solution was added to the wells for a total volume of 220 *μ*L. Samples (20 *μ*L) were loaded in the other wells, and 200 *μ*L of working reagent was added to each sample for a final reaction volume of 220 *μ*L. The solutions were mixed by briefly tapping the plate, and optical density at 405 nm was measured immediately (*t* = 0) and again after 20 min (*t* = 20 min) on a plate reader. The values were used to calculate GCase activity of the samples (U/L) based on hydrolysis of 1 *μ*M substrate/min by 1 unit of enzyme at pH 7.0.

### 2.10. Measurement of Ceramide Levels

Levels of ceramide in neuronal lysates or brain extracts were measured using a human ceramide ELISA kit (Rapidbio Biosource, West Hills, CA, USA) according to the manufacturer's instructions.

### 2.11. PP2A Activity and PLK2 Assay

PP2A activity in primary neuronal lysates was measured as previously described [36] using a PP2A colorimetric assay kit (GenMed Scientific, Arlington, VA, USA). Protein concentration was determined by the Bradford assay (GenMed Scientific) and normalized to 5 mg/mL. Activity of PLK2 in primary neuronal lysates was measured using a human PLK2 ELISA kit (Rapidbio Biosource, West Hills, CA, USA) according to the manufacturer's instructions.

### 2.12. Statistical Analysis

Data are expressed as mean ± SD. Analysis of variance (ANOVA) with Tukey's* post hoc* test and Student's *t*-test was used for comparisons among multiple groups and between 2 groups, respectively. In addition, relationships between the analytes and age, gender, and cell viability were analyzed with bivariate correlation using Pearson's correlation coefficients. Data were analyzed using SPSS v.13.0 software. *P* < 0.05 was considered statistically significant. Graphs were plotted with GraphPad Prism v.6.0 software (GraphPad Inc., La Jolla, CA, USA).

## 3. Results

### 3.1. Viability of Neurons Overexpressing *α*-syn Is Not Affected by Treatment with PD Patient Plasma

**Figure 1:**
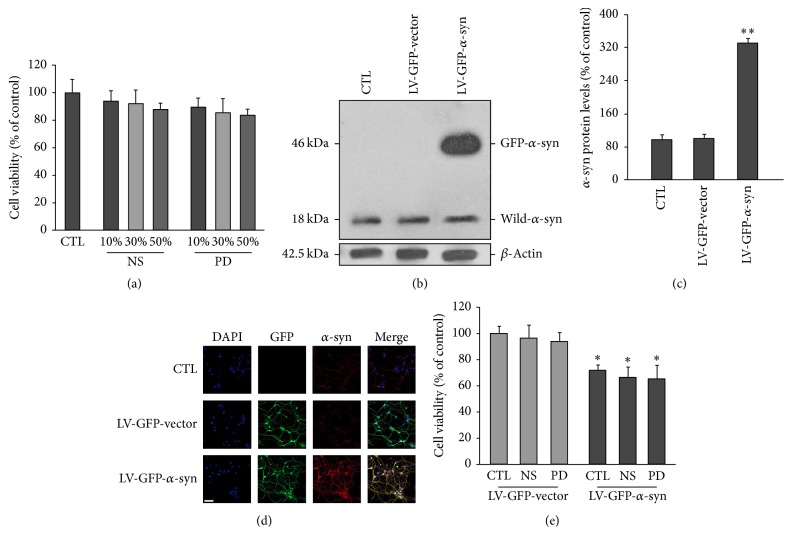
Effects of NS and PD plasma on neuronal viability. Indicated concentrations of NS and PD plasma were added to the medium of primary neuron cultures, and cell viability was evaluated 14 d later. There was no difference in viability between neurons treated with NS and PD plasma. (b) and (c) Western blot analysis of *α*-syn expression in control and vector-infected neurons. All groups expressed the same level of endogenous *α*-syn (18 kDa). Neurons infected with the LV-GFP-*α*-syn vector expressed a 46 kDa GFP-*α*-syn fusion protein. *β*-Actin was used as the loading control. The intensity of each band was normalized to that of *β*-tubulin. All values represent mean ± SEM. ^*∗∗*^ *P* < 0.01 versus control group. (d) Representative images of *α*-syn (red) and GFP (green) expression. Nuclei were counterstained with DAPI (blue). Scale bar = 50 *µ*M. (e) Neurons were infected with LV-GFP-*α*-syn or LV-GFP-vector for 3 d and then cultured in complete medium containing FBS without or with NS or PD plasma. Viability was assessed after 14 d. Neurons overexpressing *α*-syn showed reduced viability compared with controls (CTL of LV-GFP-vector). There was no difference in cell viability between neurons treated with PD or NS plasma (*n* = 12/group; one-way ANOVA followed by Tukey's* post hoc* test). ^*∗*^ *P* < 0.05 versus LV-GFP control group.

We hypothesized that alterations in the internal environment, as reflected in the blood, may contribute to *α*-syn pathology in PD. To test this, we compared the effects of NS and PD plasma on viability of neurons overexpressing *α*-syn. Primary neurons were cultured for 3 d in complete medium, which was replaced on day 4 with medium containing different concentrations (10%, 30%, and 50%) of NS and PD plasma with endogenous *α*-syn already removed. Viability was evaluated on day 14. Compared with untreated controls, neurons cultured in medium containing NS or PD plasma showed no significant viability reduction (Figure 1(a)). We then examined the effects of plasma on viability of neurons overexpressing *α*-syn. Neurons were infected with either the LV-GFP or LV-GFP-*α*-syn vector. After 24 h, medium containing NS or PD plasma was added to neuronal cultures. Western blot analysis revealed that both uninfected and infected neurons expressed low levels of endogenous *α*-syn; the band at 18 kDa corresponded to the monomeric form of the *α*-syn protein. However, neurons infected with LV-GFP-*α*-syn also expressed the 46 kDa GFP-*α*-syn fusion protein (Figures 1(b) and 1(c)). Immunocytochemical analysis revealed that all infected neurons expressed GFP, and only those infected with LV-GFP-*α*-syn had strong *α*-syn signals (Figure 1(d)), indicating that they overexpressed *α*-syn. These neurons showed reduced viability compared with those transduced with the control vector. However, there was no further reduction in cell viability in *α*-syn-overexpressing neurons treated with PD plasma compared with those that were untreated or treated with NS plasma (Figure 1(e)).

### 3.2. Viability of Neurons Treated with Extracellular *α*-syn Is Reduced by Treatment with PD Patient Plasma

**Figure 2:**
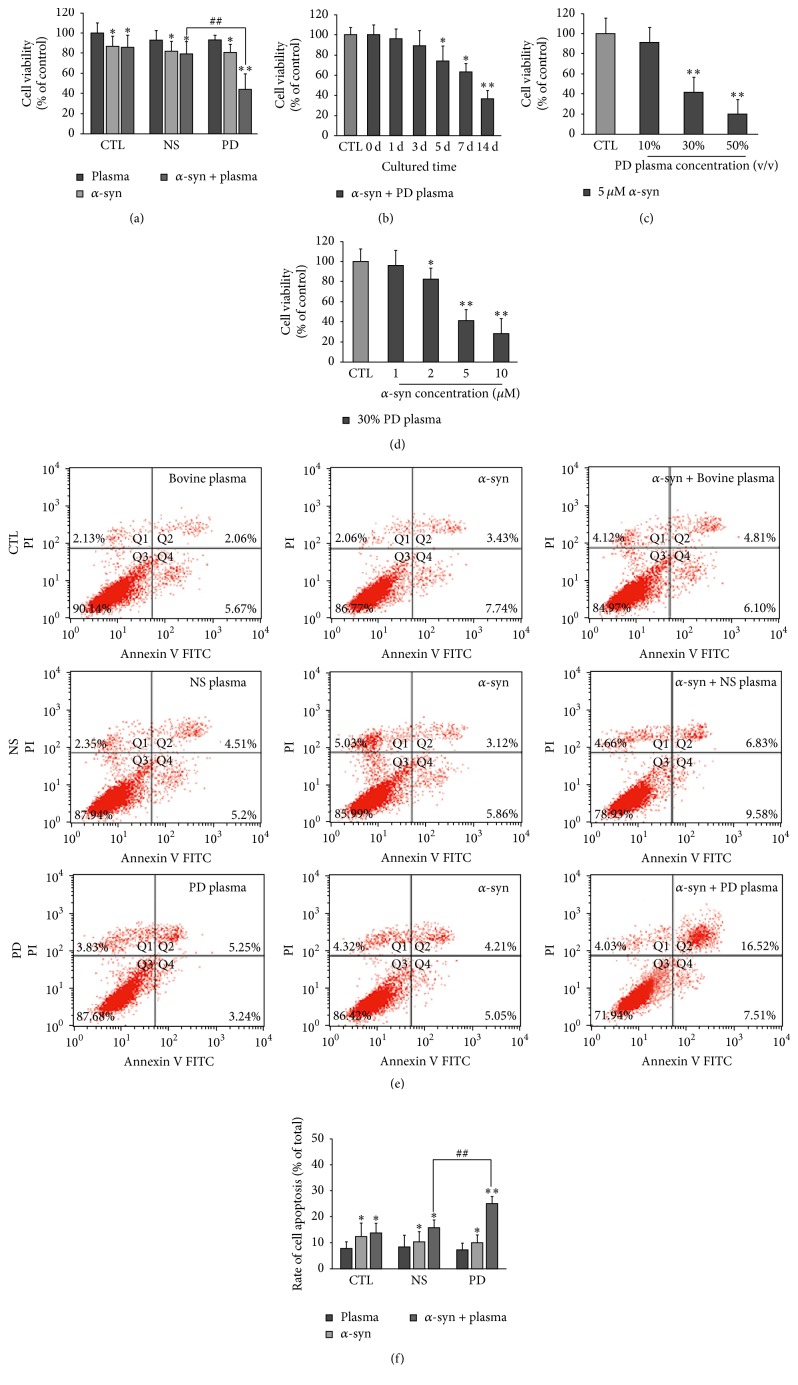
Effects of NS and PD plasma on viability of neurons treated with extracellular *α*-syn. (a) Primary neurons were cultured for 14 d in complete medium with or without 30% NS or PD plasma and 5 *µ*M *α*-syn. The control group was treated with bovine plasma. Viability of all groups was reduced in the presence of extracellular *α*-syn and was lower in neurons treated with PD as compared with NS plasma (*n* = 12; one-way ANOVA followed by Tukey's* post hoc* test). ^*∗*^ *P* < 0.05, ^*∗∗*^ *P* < 0.01 versus control group; ^##^ *P* < 0.01 versus NS group. (b) Time-dependent decrease in viability of neurons treated with 30% PD plasma and 5 *µ*M *α*-syn (*n* = 12/group; one-way ANOVA followed by Tukey's* post hoc* test). ^*∗*^ *P* < 0.05, ^*∗∗*^ *P* < 0.01 versus day 0 group. (c) Decrease in viability with increasing PD plasma concentration following treatment with 5 *µ*M *α*-syn (*n* = 12/group; one-way ANOVA followed by Tukey's* post hoc* test). ^*∗∗*^ *P* < 0.01 versus control group. (d) Decrease in viability with increasing *α*-syn concentration following treatment with 30% PD plasma (*n* = 12/group; one-way ANOVA followed by Tukey's* post hoc* test). ^*∗*^ *P* < 0.05, ^*∗∗*^ *P* < 0.01 versus day 0 group. The light gray bar in Figures 2(b), 2(c), and 2(d) refers to the average cell viability of control group in which the neurons are cultured in DMEM medium. ((e), (f)) Flow cytometry analysis of apoptotic neurons cultured with 5 *µ*M *α*-syn and treated with NS or PD plasma. Rate of apoptosis was higher in neurons treated with PD as compared with NS plasma (*n* = 5/group; one-way ANOVA followed by Tukey's* post hoc* test). ^*∗*^ *P* < 0.05, ^*∗∗*^ *P* < 0.01 versus control group; ^##^ *P* < 0.01 versus NS group.

We investigated whether PD and NS plasma differentially affected neurotoxicity of extracellular *α*-syn. To test this possibility, primary neurons were cultured for 3 d in complete medium, which was replaced on day 4 with medium containing various concentrations of recombinant *α*-syn and NS or PD plasma, followed by culture for an additional 14 d. Recombinant human *α*-syn monomers were prepared and checked with SDS-PAGE and western blot before use. All groups of neurons treated with extracellular *α*-syn (5 *µ*M) showed reduced viability compared with untreated controls (Figure 2(a)). However, neurons treated with extracellular *α*-syn plus PD plasma (30%) had reduced viability relative to those treated with extracellular *α*-syn or extracellular *α*-syn plus NS plasma, suggesting that PD plasma increased neurotoxicity of extracellular *α*-syn. Viability of neurons treated with extracellular *α*-syn plus PD plasma gradually decreased with culture time (Figure 2(b)). Furthermore, increasing the concentration of PD plasma (Figure 2(c)) or extracellular *α*-syn (Figure 2(d)) resulted in a further reduction in viability. Similarly, treatment with extracellular *α*-syn increased the rate of apoptosis relative to untreated controls, and this effect was enhanced in the presence of PD plasma (Figures 2(e) and 2(f)).

### 3.3. *α*-syn Oligomers Treated by PD Plasma Can Enter Neurons and Form Inclusions

**Figure 3:**
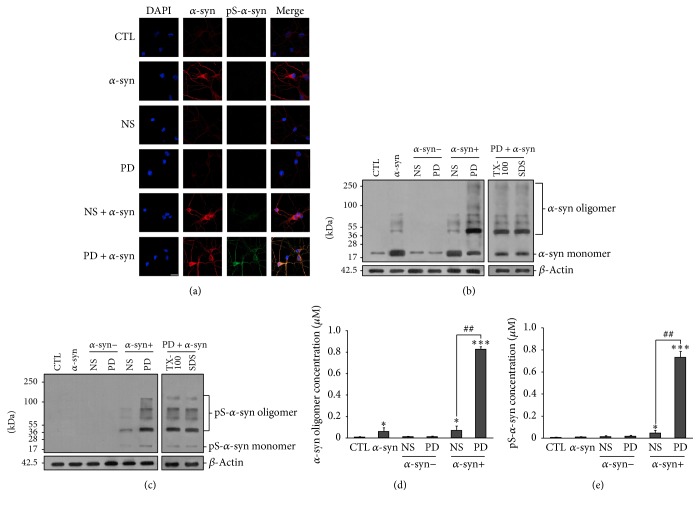
*α*-syn oligomerization and phosphorylation in neurons. (a) Immunocytochemical analysis of *α*-syn expression in neurons treated with NS or PD plasma and *α*-syn or left untreated. CTL: control neurons without treatment; *α*-syn/NS/PD: neurons treated with *α*-syn/NS plasma/PD plasma only; NS + *α*-syn or PD + *α*-syn: neurons treated *α*-syn plus NS or PD plasma. (b) Western blot analysis of total *α*-syn in the cytosol of neurons. *α*-syn oligomer levels were elevated only in neurons treated with *α*-syn plus PD plasma and were resistant to Triton X-100 and SDS. (c) Western blot analysis of cytosolic pS-*α*-syn in neurons. High levels of oligomeric pS-*α*-syn were detected in neurons treated with *α*-syn plus PD plasma, while no monomeric pS-*α*-syn was detected. ((d), (e)) Oligomeric and pS-*α*-syn detection by ELISA. Levels of both species were elevated in neurons treated with *α*-syn plus PD plasma (*n* = 5/group; one-way ANOVA followed by Tukey's* post hoc* test). ^*∗*^ *P* < 0.05, ^*∗∗∗*^ *P* < 0.001 versus control group; ^##^ *P* < 0.01 versus *α*-syn + NS group.

**Figure 4:**
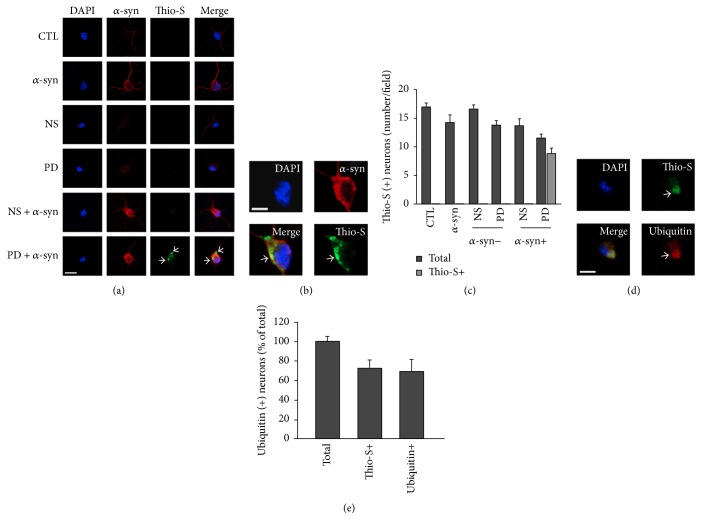
*α*-syn oligomers incubated with PD plasma accumulated in primary neurons. (a) and (b) Freshly fixed cells were double-immunostained for thioflavin S (green) and *α*-syn (red). Neurons treated with PD plasma stained positive with thioflavin S. Note the colocalization in an *α*-syn-positive and thioflavin S-positive plaque within a cytoplasmic inclusion (arrow). (a) Scale bar = 10 *µ*M. (b) Scale bar = 5 *µ*M. (c) Quantification of thioflavin S-positive neurons. Digitized images of cells were taken at 20x magnification under confocal microscopy. Images of 5 fields per dish were taken with an average of 10–20 cells per field. The number of thioflavin S-positive neurons with at least one plaque was counted for each group. Values are indicated as the number of cells per field of view. All values represent mean ± SEM, *n* = 5. (d) Primary neurons were fixed after treatment with PD plasma and *α*-syn for 14 d and then double-stained for thioflavin S and ubiquitin (red). Colocalization of thioflavin S-positive and ubiquitin-positive staining was found within a cytoplasmic inclusion (arrow) that remained following detergent extraction. Scale bar = 5 *µ*M. (e) Percentage of ubiquitin-positive neurons containing thioflavin S-positive inclusions for *α*-syn plus PD plasma group. 71.19% of neurons were found to have thioflavin S-positive plaque after being treated with *α*-syn plus PD plasma and a total of 62.71% of neurons were found colocalized with thioflavin S and ubiquitin. Mean ± SEM, *n* = 5.

*α*-syn toxicity is attributed to oligomerization, which is induced by phosphorylation [6]. We investigated whether enhanced *α*-syn toxicity by PD plasma was due to increased intracellular *α*-syn oligomerization and phosphorylation. Neurons were treated with extracellular *α*-syn and PD or NS plasma as described above, and the levels of oligomeric and phosphorylated *α*-syn in cytosolic fractions were measured. Untreated neurons showed faint fluorescent signals corresponding to *α*-syn in the immunocytochemical analysis (Figure 3(a)). In contrast, *α*-syn-treated neurons showed strong *α*-syn signals in the cell body and processes. Western blot analysis revealed low levels of endogenous *α*-syn monomers in untreated neurons (Figure 3(b)). However, in *α*-syn-treated neurons, levels of *α*-syn monomers in the cytosolic fraction were higher irrespective of treatment with PD or NS plasma. In neurons treated with *α*-syn plus PD plasma, oligomerization and phosphorylation of *α*-syn were increased in the cytosolic fraction (Figures 3(b) and 3(c)), which was confirmed by ELISA (Figures 3(d) and 3(e)). Increased *α*-syn oligomers within neurons can form fibrillary structures that lead to inclusions resembling LBs. This is the primary cause for neurodegeneration in PD [1, 2]. An important feature of the cellular inclusions is that they possessed a fibrillary structure reminiscent of LBs and other inclusions, as revealed by the fluorescent histochemical marker thioflavin S [36, 37]. Therefore, we checked whether phosphorylated *α*-syn could form inclusions within neurons. Results showed that there were thioflavin S-positive inclusions in neurons treated with *α*-syn plus PD plasma for 14 d* in vitro*. In contrast, no thioflavin S-positive staining was found in neurons of the other groups (Figures 4(a), 4(b), and 4(c)). Oligomerization of *α*-syn is the first step that leads to subsequent formation of amyloid-like fibrils. After formation of inclusions that stain positive with thioflavin S, *α*-syn oligomerizes and leads to the formation of fibrillary amyloid-like structures within the inclusions [38]. We then double-stained for thioflavin S and ubiquitin (red). Colocalization of thioflavin S-positive and ubiquitin-positive staining was found within cytoplasmic inclusions (Figures 4(d) and 4(e)). Because results demonstrated that *α*-syn aggregated more rapidly within neurons under conditions of PD plasma incubation, we conclude that extracellular *α*-syn incubated with PD plasma could enter neurons, accumulate to form pathological structures, and decrease cell viability.

### 3.4. Extracellular *α*-syn Oligomerization and Phosphorylation Are Increased by Treatment with PD Plasma

**Figure 5:**
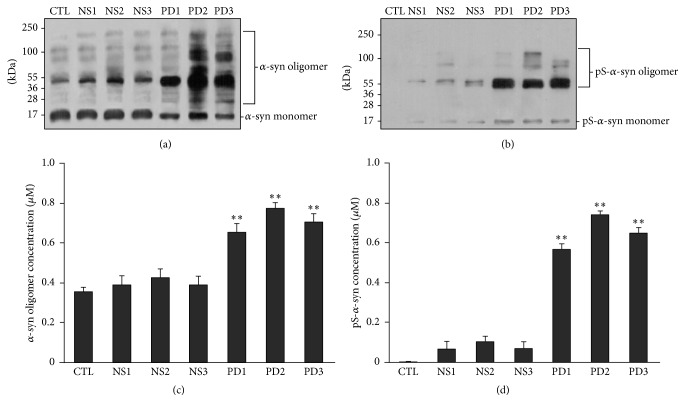
Effects of NS and PD plasma on *α*-syn oligomerization and phosphorylation in neuronal culture medium. ((a)–(d)) Recombinant human *α*-syn (5 *µ*M) was added to medium containing 30% NS or PD plasma for 14 d. Oligomeric *α*-syn and pS-*α*-syn levels were examined by western blotting ((a), (b)) and ELISA ((c), (d)) (*n* = 5/group; one-way ANOVA followed by Tukey's* post hoc* test). ^*∗*^ *P* < 0.05, ^*∗∗*^ *P* < 0.01 versus control group.

Given that PD plasma did not alter viability of primary neurons and oligomerization or phosphorylation of endogenous *α*-syn, we hypothesized that increased levels of oligomeric and phosphorylated *α*-syn observed in neurons treated with *α*-syn plus PD plasma were due to modification of the extracellular form of *α*-syn. To test this possibility, levels of oligomeric and phosphorylated *α*-syn in medium containing 5 *µ*M *α*-syn and 30% NS or PD plasma used for neuronal culture were measured. Both forms of *α*-syn were increased in medium containing *α*-syn and either NS or PD plasma as determined by western blotting and ELISA. However, the levels were higher in medium containing PD as compared with NS plasma (Figures 5(a)–5(d)).

**Figure 6:**
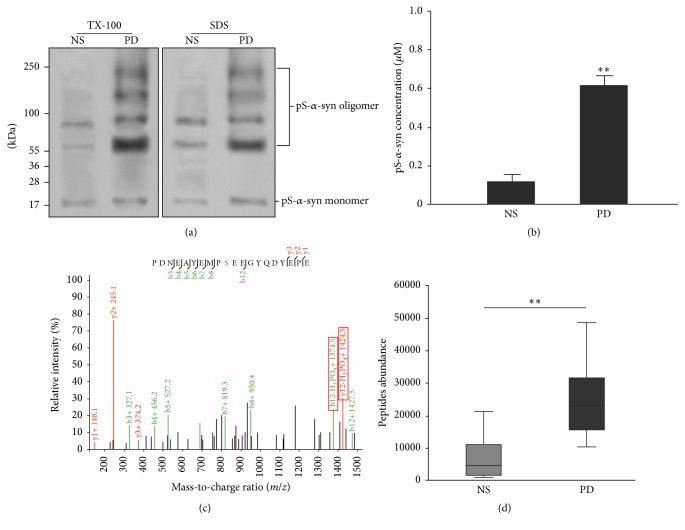
SWATCH analysis of *α*-syn phosphorylation levels following treatment with plasma. Recombinant *α*-syn was incubated with culture medium containing NS or PD plasma and then purified by immunoprecipitation. Samples were used to identify and quantify protein phosphorylation sites after checking by western blot and ELISA. (a) and (b) Results of western blot and ELISA. (c) MS analysis of phosphorylated peptides. The *α*-syn samples incubated with PD and NS plasma but not PBS were phosphorylated at Ser129. (d) Quantitative analysis of phosphorylated peptides in o-*α*-syn-NS and o-*α*-syn-PD samples. Ser129 phosphorylation increased 6.17-fold in o-*α*-syn-PD compared with 0.21-fold in o-*α*-syn-NS samples (*n* = 6/group; one-way ANOVA followed by Tukey's* post hoc* test). ^*∗∗*^ *P* < 0.01 versus NS group.

To confirm the phosphorylation status of *α*-syn in medium containing PD plasma, *α*-syn was affinity-purified and analyzed by SWATH after being checked by western blot and ELISA (Figures 6(a) and 6(b)). The ratio of phosphorylated to nonphosphorylated protein was 6.16-fold higher in the *α*-syn sample purified from medium containing PD as compared with NS plasma (Figures 6(c) and 6(d)).

### 3.5. Activities of Enzymes Regulating *α*-syn Phosphorylation and Aggregation in Neurons Treated with Extracellular *α*-syn Are Altered by PD Plasma

**Figure 7:**
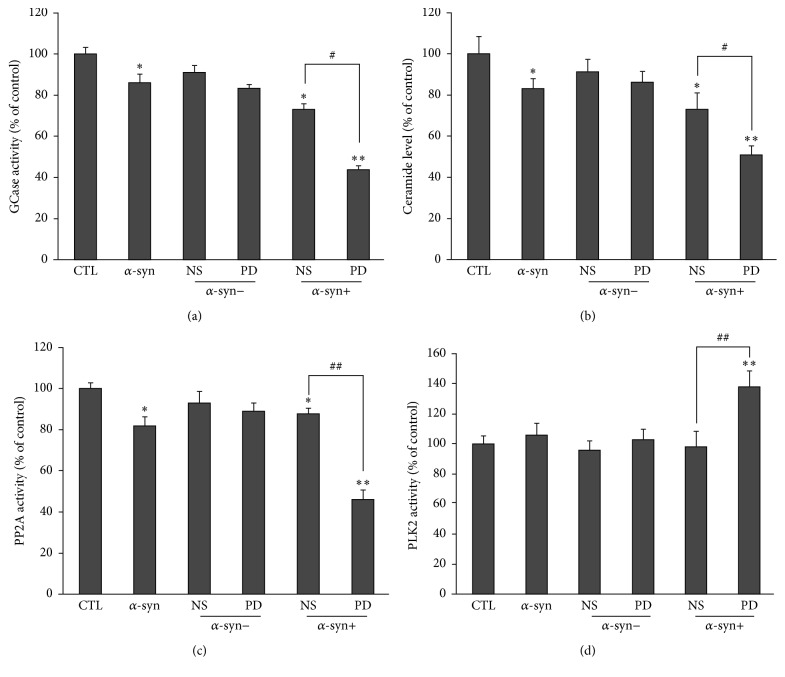
Effects of NS and PD plasma on GCase, PP2A, and PLK2 activities and ceramide levels in neurons. ((a)–(d)) Neurons were treated for 14 d and GCase activity (a), ceramide levels (b), and PP2A (c) and PLK2 (d) activities in the cytosolic fraction were evaluated (*n* = 5/group; one-way ANOVA followed by Tukey's multiple comparisons test). ^*∗*^ *P* < 0.05, ^*∗∗*^ *P* < 0.01 versus control group; ^#^ *P* < 0.05, ^##^ *P* < 0.01 versus *α*-syn + NS group.

We previously demonstrated that *α*-syn oligomers inhibit activity of GCase, an enzyme catalyzing hydrolysis of GlcCer into glucose and ceramide. This leads to a decline in ceramide levels, which in turn reduces activity of PP2A, an *α*-syn dephosphorylating enzyme. Given that neurons treated with extracellular *α*-syn plus PD plasma had increased levels of intracellular oligomeric and phosphorylated *α*-syn, we hypothesized that activities of the above enzymes as well as ceramide levels were altered in these neurons. We measured these parameters in neurons treated with NS or PD plasma or left untreated. Neurons treated with extracellular *α*-syn showed a slight reduction in GCase and PP2A activities and ceramide levels. This effect was enhanced upon treatment with extracellular *α*-syn plus NS or PD plasma, with the latter having the greatest effects (Figures 7(a)–7(c)). Moreover, the level of PLK2, which is an enzyme that promotes *α*-syn phosphorylation, was increased only in neurons treated with extracellular *α*-syn plus PD plasma and not in the other groups (Figure 7(d)).

### 3.6. Correlation between Viability of Neurons Treated with PD Plasma and Age, Gender, and Hoehn and Yahr (H & Y) Staging Scores

**Figure 8:**
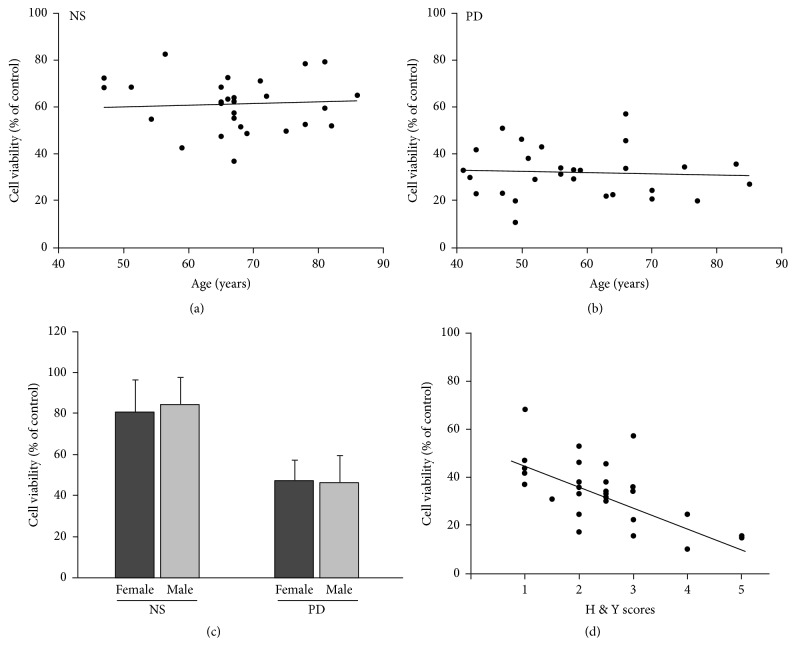
Correlation between viability of neurons treated with PD plasma and age, gender, H & Y staging scores, and PD subtype. (a) and (b) PD patients were aged 44 to 85 years, with an average age of 56.68 + 11.42 years. Viability of neurons treated with *α*-syn and PD plasma was not related to age. (a) *n* = 28, *P* > 0.05. (b) *n* = 28, *P* > 0.05. (c) The gender ratio of PD patient and NS groups was 19 : 9 (male : female). Statistical results showed that viability of neurons treated with *α*-syn and PD plasma was not related to gender. *n* = 28, *P* > 0.05. (d) Correlation between H & Y score and viability of neurons treated with plasma in the o-*α*-Syn-PD group. Cell viability decreased with increasing of H & Y score of patients. H & Y: Hoehn & Yahr. *n* = 28, *P* < 0.001.

We analyzed the correlation between neurotoxicity and age, gender, and H & Y staging scores. The results showed that neurotoxicity of *α*-syn oligomers was not related to patient age (Figures 8(a) and 8(b)) or gender (Figure 8(c)). However, neurotoxicity of *α*-syn oligomers differed according to the H & Y staging score of the patient. As the H & Y staging score increased, the effect of plasma on *α*-syn oligomers increased as well (Figure 8(d)).

## 4. Discussion

The present study demonstrated that neuronal viability was reduced and apoptosis was increased by treatment with extracellular *α*-syn plus PD plasma. Neurotoxicity was not solely due to synergism, since *α*-syn or PD plasma alone had limited effects. Therefore, extracellular *α*-syn combined with PD plasma may initiate a toxic process that leads to neuronal death. Previous studies have shown that exogenously applied *α*-syn oligomers are toxic to neurons [39]. Therefore, we hypothesized that PD plasma may increase toxicity of extracellular *α*-syn by inducing its oligomerization. Indeed, oligomeric *α*-syn levels were increased in medium containing both *α*-syn and PD plasma, and this effect was not observed with NS plasma. We hypothesize that this difference is due to modifications to *α*-syn by PD plasma. The major modification that promotes *α*-syn aggregation is phosphorylation at Ser129 [7]. Therefore, we also measured pS-*α*-syn levels in medium containing *α*-syn plus NS or PD plasma. The 2 plasma samples had distinct effects on *α*-syn phosphorylation with the latter producing higher levels of pS-*α*-syn. Moreover, all of the pS-*α*-syn in the medium was oligomerized, suggesting that *α*-syn phosphorylation at Ser129 induced by PD plasma is the major cause of its aggregation. However, we cannot rule out other modifications. A potential mechanism by which phosphorylated *α*-syn oligomers induce cell death is by directly damaging the plasma membrane, thereby increasing membrane permeability via formation of amyloid pores [30, 40]. As previously summarized, oligomerization of *α*-syn is the first step that leads to subsequent formation of amyloid-like fibrils [38]. Our results demonstrate that *α*-syn aggregates more rapidly within primary neurons under conditions of PD plasma incubation. We conclude that extracellular *α*-syn incubated with PD plasma can enter neurons, accumulate into amyloid inclusions, and decrease neuronal viability.

In addition to extracellular mechanisms, *α*-syn oligomers may exert toxic effects from inside neurons. Previous studies have indicated that *α*-syn oligomers can enter neurons [41] and cause damage by disrupting the intracellular membrane system, which includes mitochondria, the endoplasmic reticulum, Golgi complexes, and synaptic vesicles [42, 43]. In addition to these indirect effects, our results suggest that *α*-syn oligomers can directly inhibit activity of GCase [35], which leads to accumulation of more *α*-syn oligomers since GCase is responsible for autophagic degradation of *α*-syn oligomers [44]. Moreover, inhibition of GCase activity by *α*-syn oligomers may decrease production of ceramide, a natural activator of PP2A, which promotes *α*-syn phosphorylation and aggregation. Indeed, we found that neurons treated with extracellular *α*-syn plus PD plasma had higher levels of intracellular phosphorylated and oligomerized *α*-syn and lower GCase and PP2A activities and ceramide levels. We also observed an increase in activity of PLK2, an enzyme that promotes *α*-syn phosphorylation [12], although the relationship between this enzyme and *α*-syn oligomerization is unclear. It is interesting that the above changes have been observed in the brains of PD patients, indicating that these processes contribute to development and progression of PD.

Furthermore, neurotoxicity of *α*-syn incubated with PD plasma was positively correlated with H & Y staging scores. As H &Y staging score is an important index reflecting the movement ability and disease progression of PD patients, these results indicate that the abnormal of ability of PD plasma to modify *α*-syn may be an important factor in progressive neurodegeneration of PD.

The results of the present study provide insight into the mechanisms underlying the spread of *α*-syn pathology. *α*-syn is secreted into the extracellular space from neurons, especially those with intracellular *α*-syn accumulation [31]. This may represent a new pathway through which *α*-syn pathology spreads and one of the reasons which lead to disease aggravation in some patients.

## 5. Conclusions

The present study demonstrates that PD plasma increases extracellular *α*-syn neurotoxicity by promoting its phosphorylation and oligomerization. In addition, the study provides evidence that phosphorylated *α*-syn oligomers enter neurons and accumulate rapidly, which induces a series of metabolic changes including activation of PLK2, inhibition of GCase and PP2A, and reduction of ceramide levels. Furthermore, neurotoxicity is correlated with the H & Y staging scores of patients.

## Figures and Tables

**Table 1:** Population characteristics of control group and PD patient group.

	Age, yr	Gender, F/M	PD duration, yr	LEDD, mg	MoCA, mean ± SD (range)	UPDRS III	BDI
CTL, *n* = 28	55.41 ± 10.23	9/19	—	—	26.5 ± 1.4 (25–30)	—	6 ± 3
PD, *n* = 28	56.68 ± 11.42	9/19	4.88 ± 3.6	506.25 ± 260.45	25.8 ± 1.6 (24–30)	33.25 ± 12.14	7 ± 3

M = male; F = female; PD = Parkinson disease patients; LEDD = meanl-dopaequivalent daily dose; MoCA = Montreal Cognitive Assessment; UPDRS III = Unified Parkinson Disease Rating Scale, part III (motor); BDI = Beck Depression Inventory.

## Abbreviations

*α*-syn: *α*-Synuclein
DDA: Data-dependent acquisition
ELISA: Enzyme-linked immunosorbent assay
GCase: Glucocerebrosidase
GFP: Green fluorescent protein
LB: Lewy body
MS/MS: Tandem mass spectrometry
o-*α*-syn: Oligomers of *α*-synuclein
PD: Parkinson's disease
PLK2: Polo-like kinase 2
PP2A: Protein phosphatase 2A
pS-*α*-syn: *α*-Synuclein phosphorylated at Ser129
pS-o-*α*-syn: Oligomers of *α*-synuclein phosphorylated at Ser129
SWATH: Sequential window acquisition of all theoretical mass spectra.
